# Five-Year Outcomes of Omidenepag Isopropyl Monotherapy for Untreated Primary Open-Angle Glaucoma

**DOI:** 10.7759/cureus.98038

**Published:** 2025-11-28

**Authors:** Akiyasu Kanamori, Noriko Kanamori

**Affiliations:** 1 Department of Ophthalmology, Kanamori Eye Clinic, Akashi, JPN; 2 Department of Ophthalmology, Kobe University, Kobe, JPN

**Keywords:** glaucoma, intraocular pressure, omidenepag isopropyl, prostaglandin ep2, treatment continuation

## Abstract

Background

Omidenepag isopropyl (OMDI) eye drops are newly developed for glaucoma treatment. OMDI is a non-prostaglandin EP2 agonist with a mechanism of action distinct from other commonly used drugs, such as prostaglandin FP agonists. The aim of this study was to evaluate the long-term efficacy and safety of OMDI administered as first-line therapy for untreated primary open-angle glaucoma (POAG) over five years.

Methods

This retrospective study included one eye per patient. A total of 103 treatment-naïve, consecutive POAG eyes newly administered OMDI between December 2018 and March 2020 were included. Reasons for discontinuing OMDI due to adverse effects or other factors were analyzed. The primary outcome was change in intraocular pressure (IOP) over time, and secondary outcomes were visual field progression and rate of persistence with OMDI monotherapy. Pre- and posttreatment IOP and mean deviation (MD) in Humphrey visual field testing were analyzed using a mixed-effects model for repeated measures in IBM SPSS Statistics for Windows, Version 25.0 (Released 2017; IBM Corp., Armonk, NY, USA), with statistical significance set at p < 0.05. Kaplan-Meier analysis was performed to evaluate the persistence of OMDI monotherapy.

Results

Among 103 initial eyes, 13 cases were discontinued early (up to six months) due to adverse events (e.g., conjunctival hyperemia), and nine cases were lost to follow-up (e.g., transferred to another institution). In the remaining 81 eyes, over the five-year follow-up, 20 eyes (25%, 20/81) required additional glaucoma medications due to inadequate efficacy associated with visual field progression or structural changes. Two eyes underwent cataract surgery (one eye cataract surgery alone, one eye combined cataract surgery and microhook trabeculotomy). Median (IQR) IOP decreased significantly from baseline to one, two, three, four, and five years: 17 (15.5-18.5), 15 (13-16), 14 (13-15), 14 (13-16), 15 (13-16), and 14 (13-16) mmHg, respectively (p = 0.008, mixed-effects model). Five years after initiating OMDI, monotherapy remained feasible in 73% of cases (59/81). The visual field of these 59 eyes under OMDI monotherapy changed from -1.47 (-0.48 to -2.48) dB at baseline to -0.94 (-0.34 to -3.15) dB at five years, with no significant change in MD values (p = 0.34, mixed-effects model). No cases discontinued OMDI due to adverse events such as allergies after six months of treatment.

Conclusions

OMDI achieved sustained IOP reduction and demonstrated good tolerability over five years. This retrospective study suggests that OMDI may be a reliable first-line treatment option for glaucoma. These findings require confirmation in prospective multicenter trials.

## Introduction

Glaucoma is a chronic, progressive optic neuropathy characterized by the loss of retinal ganglion cells and irreversible deterioration of the visual field. The only proven method to delay this loss is the sustained reduction of intraocular pressure (IOP). Prostaglandin F (FP) receptor agonists, such as latanoprost and travoprost, are the primary first-line topical therapies due to their potent IOP-lowering effects and the convenience of once-daily dosing. However, chronic exposure to prostaglandin agonists commonly induces prostaglandin-associated periorbitopathy (PAP), a condition involving various periocular changes such as deepening of the upper eyelid sulcus (DUES), loss of periorbital fat, enophthalmos, and ptosis. These changes can be cosmetically concerning [1,2].

Recovery of DUES after modifying prostaglandin therapy has been documented, indicating that some FP-mediated tissue changes are reversible. Several single-center switching studies have reported improvements in PAP after converting from FP agonists to alternative agents, such as omidenepag isopropyl (OMDI) [3]. This finding underscores the clinical importance of selecting an agent with a non-FP mechanism when periocular appearance is a concern. Importantly, the severity of PAP has been associated with poorer surgical outcomes following filtration procedures [4,5].

OMDI (0.002% OMDI ophthalmic solution, Eybelis^®^) is a selective prostanoid EP2 receptor agonist approved for reducing IOP. OMDI increases aqueous outflow through both the uveoscleral and trabecular pathways. Randomized controlled trials have demonstrated that OMDI reduces IOP similarly to latanoprost in the short term [6]. OMDI has distinct safety features, including fewer pigmentary and eyelid changes but more frequent, generally mild, conjunctival hyperemia. In the RENGE study, OMDI produced clinically meaningful IOP reductions over 12 months [7]. Phase 3 and targeted studies, including those involving latanoprost non- or low-responders, have further characterized the efficacy of OMDI across populations [8,9].

A systematic aggregation of these datasets (i.e., a meta-analysis) shows consistent mean IOP reductions with OMDI across trials and observational studies [10,11]. Real-world, multicenter observational cohorts, including three-year multicenter data, have corroborated trial results, demonstrating sustained IOP reductions and acceptable tolerability in East Asian populations, where normal-tension glaucoma is prevalent [12].

Previously, we reported one-year, single-center outcomes of OMDI in eyes with primary open-angle glaucoma (POAG), showing a mean IOP decrease from 17.1 mmHg to 13.9 mmHg, along with acceptable short-term tolerability [13]. However, published long-term (>3 years) real-world evidence for first-line OMDI monotherapy is limited. To address this gap, the present study evaluates five-year, real-world outcomes of OMDI monotherapy in treatment-naïve POAG patients, focusing on sustained IOP control, treatment continuation, and ocular safety.

## Materials and methods

This retrospective observational study was conducted at Kanamori Eye Clinic in Kobe, Japan. The study protocol adhered to the tenets of the Declaration of Helsinki and was approved by the Kanamori Eye Clinic Ethics Committee. Patients with newly diagnosed POAG who began OMDI monotherapy between December 2018 and March 2020 were included. OMDI was administered once daily at the start of the monocular trial. Only phakic eyes were eligible.

Glaucoma was diagnosed based on optic disc changes, visual field defects, and open angles on gonioscopy. None of the patients had received prior glaucoma treatments, including topical medications or laser surgery. Eyes with secondary glaucoma, prior ocular surgery (other than uncomplicated cataract extraction), ocular trauma, uveitis, or systemic conditions affecting IOP were excluded. One eye per patient was included to minimize inter-eye correlation. When both eyes received OMDI, the eye with the higher baseline IOP was analyzed; if IOP was equal in both eyes, the first-treated eye was enrolled.

Follow-up data were extracted from routine visits, typically performed at one week, one month, and every three to six months thereafter. IOP was measured using Goldmann applanation tonometry during office hours (9 a.m. to 7 p.m.) by a single physician (AK) to minimize diurnal fluctuation. Clinical data, including age, sex, IOP, uncorrected and best-corrected visual acuity (BCVA), mean deviation (MD) from the 24-2 SITA-standard protocol using the Humphrey Field Analyzer (Carl Zeiss Meditec, Dublin, CA, USA), and adverse events were collected retrospectively from medical records. Reasons for discontinuation, addition of therapy, or transfer were also recorded. Adverse events were identified from clinician notes and patient-reported symptoms documented in electronic medical records.

The primary outcome was the change in IOP over time. Secondary outcomes were visual field MD and the rate of continuation as OMDI monotherapy. Pre- and posttreatment IOP and Humphrey visual field MD were analyzed using a mixed-effects model for repeated measures in IBM SPSS Statistics for Windows, Version 25.0 (Released 2017; IBM Corp., Armonk, NY, USA), with statistical significance set at p < 0.05. IOP and MD values are expressed as median and IQR.

All statistical analyses were performed using IBM SPSS Statistics for mixed-effects modeling or MedCalc Statistical Software version 20.010 (MedCalc Software Ltd., Ostend, Belgium) for other analyses. P-values < 0.05 were considered statistically significant. Kaplan-Meier analysis was used to assess persistence of OMDI monotherapy, with an event defined as either discontinuation of OMDI or addition of any adjunctive antiglaucoma medication.

## Results

The baseline characteristics of the 103 initial eyes are shown in Table 1.

**Table 1:** Baseline characteristics of 103 initial eyes (median and IQR) IOP, intraocular pressure; MD, mean deviation

Parameter	Value
Age (years)	56 (49.5-64)
Sex (M/F)	48/53
Baseline IOP (mmHg)	16.5 (15.5-18.5)
MD in HFA 24-2 (dB)	-5 (-2.25 to -7.5)
Central corneal thickness (µm)	543 (520-574)

During the early phase (within six months), adverse reactions occurred in 13 patients (11.0%) (Table 2). One eye experienced a decrease in uncorrected visual acuity due to increasing myopia (1 D). OMDI administration was discontinued in these patients, and all adverse reactions resolved. Four patients showed insufficient IOP reduction, defined as a reduction rate below 10% under physician judgment. Nine patients discontinued follow-up for other reasons (Table 2).

**Table 2:** Reasons for discontinuation or treatment modification IOP, intraocular pressure

Specific reason	N (%)
Insufficient IOP	4 (3.9%)
Adverse events	
Conjunctival hyperemia	3 (2.9%)
Blepharitis	1 (1%)
Iritis	2 (1.9%)
Decrease visual acuity	1 (1%)
Photophobia	1 (1%)
Blurred vision	1 (1%)
Loss to follow-up	
Hospital transfer	3 (2.9%)
Unknown	3 (2.9%)
Pregnancy	1 (1%)
Death	1 (1%)
Deterioration of general condition	1 (1%)

Table 3 presents the baseline characteristics of the remaining 81 eyes after excluding early dropouts and adverse events. The median (IQR) age was 56 (50-63) years, and the median baseline IOP was 17 (15.5-18.5) mmHg.

**Table 3:** Baseline characteristics of patients (N = 81; median and IQR) IOP, intraocular pressure; MD, mean deviation

Parameter	Value
Age (years)	56 (50-63)
Sex (M/F)	38/43
Baseline IOP (mmHg)	17 (15.5-18.5)
MD in HFA 24-2 (dB)	-1.47 (-0.48 to -2.48)
Central corneal thickness (µm)	547 (520-575)

**Figure 1:**
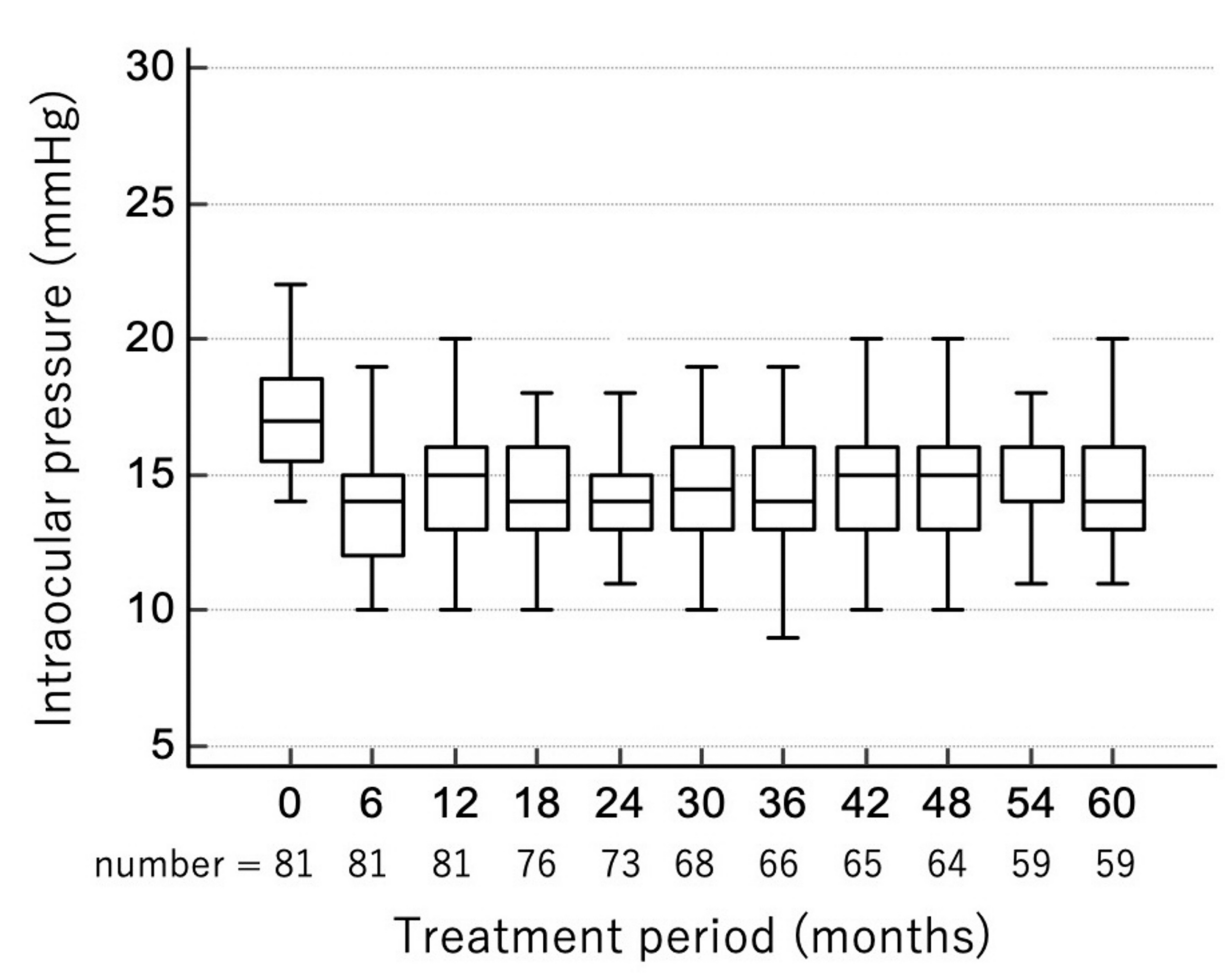
Box-and-whisker plots showing IOP (mmHg) before and during OMDI monotherapy over 60 months (median and IQR) IOP, intraocular pressure; OMDI, omidenepag isopropyl

As shown in Figure 1, median (IQR) IOP decreased significantly from baseline to one, two, three, four, and five years: 17 (15.5-18.5), 15 (13-16), 14 (13-15), 14 (13-16), 15 (13-16), and 14 (13-16) mmHg, respectively (p = 0.008, mixed-effects model). These results indicate that OMDI monotherapy provided sustained and clinically meaningful IOP reduction over five years, without evidence of tachyphylaxis.

After five years, 59 of 81 eyes (73%) were controlled with OMDI monotherapy alone. Twenty-two eyes (27%) required additional therapy during the five-year period at a median time of 24 months (range: 18.75-32 months). Twenty eyes (25%, 20/81) required additional glaucoma eye drops due to inadequate efficacy associated with visual field progression or structural changes. Among these, 10 eyes received carteolol sustained-release, eight eyes received brimonidine, one eye received brinzolamide, and one eye received a combination of brimonidine and brinzolamide.

**Figure 2:**
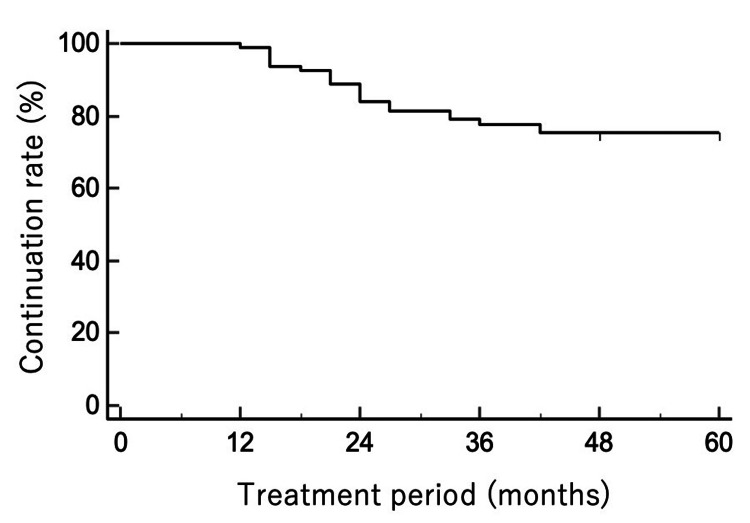
Kaplan-Meier curve showing continuation of OMDI monotherapy over 60 months An event was defined as treatment discontinuation or addition of adjunctive therapy. OMDI, omidenepag isopropyl

Two eyes underwent cataract surgery: one eye had cataract surgery alone at 54 months due to cataract progression, and another underwent combined cataract surgery and microhook trabeculotomy at 24 months due to worsening cataract and glaucoma. OMDI was discontinued in these eyes after surgery. Kaplan-Meier analysis showed cumulative persistence rates of OMDI monotherapy (Figure 2).

No cases of PAP, iris pigmentation, or eyelash changes were observed during the five-year period, as evaluated by slit-lamp examination. No cases discontinued OMDI due to adverse events such as allergies after six months of treatment. All eyes maintained BCVA of at least 20/20 at baseline and throughout the five-year period.

**Figure 3:**
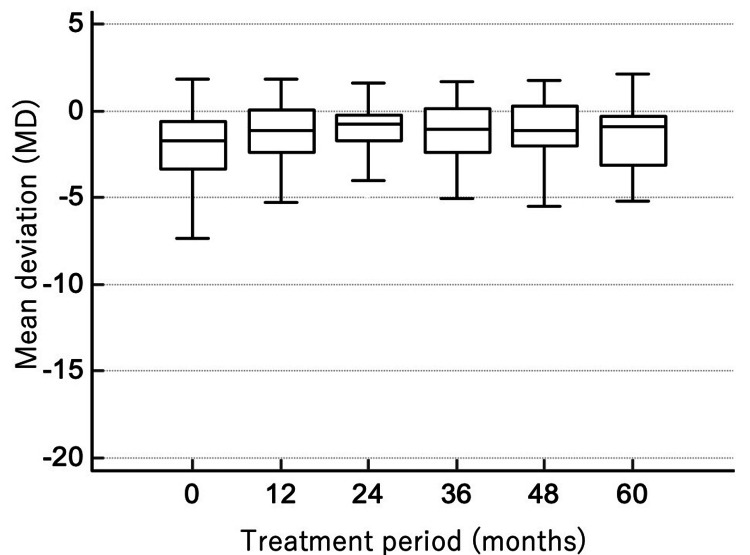
Box-and-whisker plots showing MD (dB) over time in eyes receiving OMDI monotherapy (median and IQR) MD, mean deviation; OMDI, omidenepag isopropyl

The visual fields of the 59 eyes under OMDI monotherapy for five years are shown in Figure 3. MD at baseline was -1.47 (-0.48 to -2.48) dB and -0.94 (-0.34 to -3.15) dB at five years. The change in MD over five years was not statistically significant (p = 0.34), indicating stable visual fields during OMDI monotherapy.

## Discussion

This is the first five-year real-world analysis of OMDI monotherapy in treatment-naïve POAG eyes. Previous clinical trials and observational studies have demonstrated similar IOP-lowering effects over shorter periods. In the RENGE trial, OMDI achieved clinically meaningful reductions over 12 months, with subgroup analyses reporting decreases of approximately 3-6 mmHg depending on baseline IOP [7]. The AYAME Phase 3 study likewise documented non-inferiority versus latanoprost, with early IOP reductions of around 5-6 mmHg [6]. In latanoprost low/nonresponder cohorts, OMDI produced additional IOP reductions of approximately 4-5 mmHg [8]. Meta-analyses of clinical and observational data reported a pooled mean IOP reduction of -4.68 mmHg, confirming OMDI’s consistent efficacy across diverse settings [10,11]. Three-year multicenter data from Inoue et al. demonstrated a mean IOP reduction from 15.7 to 13.2 mmHg in normal-tension glaucoma patients [12]. Excluding cases with reasons such as hospital transfer or inaccurate IOP measurements, OMDI achieved treatment persistence for three years in 66% (48/73) of cases.

The five-year outcomes of the present study (17.1 mmHg to 14.7 mmHg) confirm stable, long-term IOP reduction. Furthermore, after initial early-phase side effects, 73% (59/81) of cases continued OMDI monotherapy for five years. The absence of attenuation in the IOP-lowering effect contrasts with historical experience using β-blockers, where timolol demonstrated “long-term drift” [14]. Overall, accumulating data demonstrate that OMDI provides sustained IOP reduction with excellent tolerability in both short- and long-term treatment.

Safety outcomes were favorable. The most frequent early adverse event was mild conjunctival hyperemia, which resolved after OMDI discontinuation. Conjunctival hyperemia is among the most commonly reported adverse reactions associated with topical ocular hypotensive agents and is cosmetically concerning. In a post-marketing observational analysis up to 12 months, pretreatment incidence of hyperemia was 9.5%; this increased to 16.6% at three months posttreatment but decreased thereafter, returning to baseline at six months and beyond [15]. Recent objective analyses indicate that the frequency, severity, and duration of hyperemia induced by OMDI are nearly equivalent to those of conventional FP analogues [16]. In retrospective cohort studies conducted at our center and others, persistence rates for OMDI monotherapy were 80% at six months and 78% at 12 months [17]. The primary reason for discontinuation was insufficient IOP reduction (~50%), followed by conjunctival hyperemia (~19%), most of which occurred within the first month of treatment [17]. These findings underscore the importance of the early treatment phase in determining long-term continuation.

No cases of iritis or cystoid macular edema were observed in the late phase, consistent with prior Phase 3 studies [7,9]. A major advantage of OMDI is the absence of typical FP-mediated periocular remodeling. FP agonists are well known to cause PAP in a significant proportion of chronic users, and recovery of DUES has been demonstrated after switching to OMDI [3]. In our five-year monotherapy cohort, no PAP was observed during routine clinical consultation, suggesting that OMDI is well-tolerated and rarely requires discontinuation beyond the initial adaptation period.

Limitations of this study include its retrospective single-center design and modest sample size, although the five-year follow-up strengthens its clinical relevance. Prospective multicenter trials are warranted to confirm these findings. PAP in this study was assessed clinically rather than through strict facial photographic evaluation; future prospective studies using standardized imaging methods may yield more reliable results.

## Conclusions

This five-year real-world study provides the longest reported follow-up for OMDI monotherapy, confirming its sustained IOP-lowering effect, long-term stability, and excellent tolerability. No PAP was observed, highlighting its safety for phakic glaucoma patients. These findings support the use of OMDI as a reliable first-line treatment, particularly for patients concerned with preserving periocular appearance, and offer valuable real-world evidence for its long-term efficacy and safety in managing POAG.

## References

[1] Liu C, Wong T, Leung D (2024). Clinical staging of prostaglandin-associated periorbitopathy syndrome in glaucoma: a review from Asia. Semin Ophthalmol.

[2] Sakata R, Shirato S, Miyata K, Aihara M (2013). Recovery from deepening of the upper eyelid sulcus after switching from bimatoprost to latanoprost. Jpn J Ophthalmol.

[3] Oogi S, Nakakura S, Terao E, Fujisawa Y, Tabuchi H, Kiuchi Y (2020). One-year follow-up study of changes in prostaglandin-associated periorbital syndrome after switch from conventional prostaglandin F2alfa to omidenepag isopropyl. Cureus.

[4] Ishida A, Miki T, Naito T, Ichioka S, Takayanagi Y, Tanito M (2023). Surgical results of trabeculectomy among groups stratified by prostaglandin-associated periorbitopathy severity. Ophthalmology.

[5] Yoshida Y, Harano A, Miki T (2025). Impact of prostaglandin-associated periorbitopathy on surgical outcomes of trabeculectomy and ahmed glaucoma valve implantation. Graefes Arch Clin Exp Ophthalmol.

[6] Aihara M, Lu F, Kawata H, Iwata A, Odani-Kawabata N, Shams NK (2020). Omidenepag isopropyl versus latanoprost in primary open-angle glaucoma and ocular hypertension: the Phase 3 AYAME study. Am J Ophthalmol.

[7] Aihara M, Lu F, Kawata H, Iwata A, Odani-Kawabata N (2021). Twelve-month efficacy and safety of omidenepag isopropyl, a selective EP2 agonist, in open-angle glaucoma and ocular hypertension: the RENGE study. Jpn J Ophthalmol.

[8] Aihara M, Ropo A, Lu F, Kawata H, Iwata A, Odani-Kawabata N, Shams N (2020). Intraocular pressure-lowering effect of omidenepag isopropyl in latanoprost non-/low-responder patients with primary open-angle glaucoma or ocular hypertension: the FUJI study. Jpn J Ophthalmol.

[9] Panarelli JF, Bowden EC, Tepedino ME, Odani-Kawabata N, Pei Z, McLaurin EB, Ropo A (2023). Omidenepag isopropyl in latanoprost low/nonresponders with primary open-angle glaucoma or ocular hypertension: a phase 3, nonrandomized, two-phase, open-label study. J Glaucoma.

[10] Sharma S, Singh R, Balas M, Mathew DJ (2025). Safety and efficacy of omidenepag isopropyl for elevated intraocular pressure: a systematic review and meta-analysis. Am J Ophthalmol.

[11] Kuo HT, Yeh CY, Hsu AY, Ho JH, Lin CJ, Tsai YY (2023). Clinical efficacy of omidenepag isopropyl for primary open-angle glaucoma, normal tension glaucoma, or ocular hypertension: a meta-analysis. J Ocul Pharmacol Ther.

[12] Inoue K, Shiokawa M, Kunimatsu-Sanuki S, Kang J, Uraki T, Tomita G, Ishida K (2024). Three-year efficacy and safety of omidenepag isopropyl in patients with normal tension glaucoma. Jpn J Ophthalmol.

[13] Kanamori A, Wakabayashi S, Kanamori N (2021). One-year real-world results of omidenepag isopropyl monotherapy in treatment-naïve open-angle glaucoma [Article in Japanese]. Rinsho Ganka (Jpn J Clin Ophthalmol).

[14] Boger WP 3rd, Puliafito CA, Steinert RF, Langston DP (1978). Long-term experience with timolol ophthalmic solution in patients with open-angle glaucoma. Ophthalmology.

[15] Nakazawa T, Takahashi K, Kuwayama Y, Nomura A, Shimada F (2022). Interim results of post-marketing observational study of omidenepag isopropyl for glaucoma and ocular hypertension in Japan. Adv Ther.

[16] Tokumo K, Yoneda T, Nakaniida Y (2025). Objective hyperemia and intraocular pressure changes following omidenepag isopropyl application. PLoS ONE.

[17] Nakakura S, Kanamori A, Fukuma Y, Wakabayashi S, Nagata Y, Adachi M (2021). Evaluation of early medication persistence with omidenepag isopropyl, a topical selective prostaglandin EP2 agonist, in patients with glaucoma: a retrospective two-institute study. BMJ Open.

